# Construction of *Corynebacterium glutamicum* cells as containers encapsulating dsRNA overexpressed for agricultural pest control

**DOI:** 10.1007/s00253-019-10113-9

**Published:** 2019-09-05

**Authors:** Shuhei Hashiro, Mayu Mitsuhashi, Yasuhiko Chikami, Haruka Kawaguchi, Teruyuki Niimi, Hisashi Yasueda

**Affiliations:** 1 Institute for Innovation, Ajinomoto Co., Inc., 1-1, Suzuki-cho, Kawasaki-ku, Kawasaki, 210-8681 Japan; 2 Division of Evolutionary Developmental Biology, National Institute for Basic Biology, Nishigonaka 38, Myodaiji, Okazaki, Aichi 444-8585 Japan; 3 Department of Basic Biology, School of Life Science, SOKENDAI (The Graduate University for Advanced Studies), Nishigonaka 38, Myodaiji, Okazaki, Aichi 444-8585 Japan; 4 Research and Development Center for Precision Medicine, University of Tsukuba, 1-2, Kasuga, Tsukuba-shi, Ibaraki 305-8550 Japan

**Keywords:** RNA-based pesticide, dsRNA production, *Corynebacterium glutamicum*, *Henosepilachna vigintioctopunctata*, Inhibitor of apoptosis, Pest control

## Abstract

Double-stranded RNA (dsRNA) inducing RNA interference (RNAi) is expected to be applicable to management of agricultural pests. In this study, we selected a ladybird beetle, *Henosepilachna vigintioctopunctata*, as a model target pest that devours vegetable leaves, and examined the effects of feeding the pest sterilized microbes highly accumulating a target dsRNA for RNAi induction. We constructed an efficient production system for diap1*-dsRNA, which suppresses expression of the essential gene *diap1* (encoding death-associated inhibitor of apoptosis protein 1) in *H. vigintioctopunctata*, using an industrial strain of *Corynebacterium glutamicum* as the host microbe. The diap1*-dsRNA was overproduced in *C. glutamicum* by convergent transcription using a strong promoter derived from corynephage BFK20, and the amount of dsRNA accumulated in fermented cells reached about 75 mg per liter of culture. In addition, we developed a convenient method for stabilizing the dsRNA within the microbes by simply sterilizing the diap1*-dsRNA-expressing *C. glutamicum* cells with ethanol. When the sterilized microbes containing diap1*-dsRNA were fed to larvae of *H. vigintioctopunctata*, *diap1* expression in the pest was suppressed, and the leaf-feeding activity of the larvae declined. These results suggest that this system is capable of producing stabilized dsRNA for RNAi at low cost, and it will open a way to practical application of dsRNA as an environmentally-friendly agricultural insecticide.

**Electronic supplementary material:**

The online version of this article (10.1007/s00253-019-10113-9) contains supplementary material, which is available to authorized users.

## Introduction

Many powerful chemical pesticides have been used to combat pests in agriculture. Although they have brought great benefits to crop cultivation, they also have various undesirable environmental consequences, for example, effects on beneficial insects and soil microbes and the emergence of pesticide-resistant pests (Woodcock et al. 2016; Alyokhin et al. 2008). Thus, the development of environmentally-friendly pest control methods that replace conventional chemical pesticides is desired and required. Recently, RNA has attracted attention as a potential candidate for pest control. When RNA interference (RNAi) by double-stranded RNA (dsRNA) is induced with respect to a gene essential for the growth of a target pest, expression of that gene is suppressed, and, as a result, the growth of the pest is inhibited. Thus, RNA molecules having such a function can be called RNA-based pesticides or RNA insecticides (Palli 2014; Gu and Knipple 2013).

The ladybird beetle *Henosepilachna vigintioctopunctata* is an agricultural pest, since the insect exclusively feeds on leaves of *Solanaceae* crops such as potato, tomato, eggplant, and so on. It is one of the most destructive pests found in Japan, China, India, and other countries and causes considerable economic damage, especially in vegetable cultivation (Wang et al. 2018; Jeyasankar et al. 2014). The pest becomes the adult from pupa after four molting stages of larvae (Venkatesha 2006), and both larvae and adults are known to eat the epidermal tissues of vegetable leaves greedily. Thus, we selected *H*. *vigintioctopunctata* as a model pest for targeting by RNA-based pesticide, and we have been studying the effect of dsRNA for RNAi on this pest. In previous work, we indicated that the leaf-eating activity of *H*. *vigintioctopunctata* could be efficiently suppressed by feeding the insect dsRNA corresponding to part of the *diap1* (death-associated inhibitor of apoptosis protein 1) gene (Niimi et al. 2015; Chikami et al. 2019). The *diap1* gene product interacts with the programmed cell death (PCD)-caspases and plays a role in proper function of the PCD process (Kumar and Cakouros 2004), which is particularly important in the metabolism of insects; disruption of DIAP1 function induces cell death. The dsRNA used in the previous study was prepared by in vitro enzymatic synthesis using a *diap1*-cDNA fragment which we originally cloned from *H*. *vigintioctopunctata* (Niimi et al. 2015).

So far, growth inhibition of various insects (crop pests, ants, termites, and so on) through RNAi action by dsRNA has been reported (Zhou et al. 2008; Katoch et al. 2013; Bolognesi et al. 2012; Christiaens et al. 2016; Sun Miguel and Scott 2016; Knorr et al. 2018; Tariq et al. 2019). The preparation method of such dsRNAs was exclusively based on an in vitro T7 transcription system or a similar system using the T7 RNA polymerase/promoter combination in the host microbe *Escherichia coli* (Zhu et al. 2011; Ratzka et al. 2013; Tenllado et al. 2003). However, these methods are not fully satisfactory in terms of manufacturing cost and robustness in mass production of target dsRNAs. Reduction of the production cost of dsRNA is the first crucial challenge in its practical application in agriculture.

In previous study, we have succeeded in overproducing recombinant RNA molecules in *Corynebacterium glutamicum* having an efficient RNA expression system equipped with strong promoter F1, derived from corynephage BFK20 (Hashiro et al. 2019a, b). Although the model RNA overexpressed was single-stranded (ss) with double-stranded structures in part, accumulation of the target RNA reached about 300 mg per liter of culture. *C. glutamicum* is well known as a microbe capable of overproducing amino acids at very low cost. The strain is nonpathogenic, safe, robust in large-scale fermentation, and its use benefits from the experience of industrial application for decades (Ikeda and Takeno 2013; Yasueda 2014; Lee and Wendisch 2017). Therefore, based on our *C. glutamicum* RNA expression system, we aimed to construct an efficient preparation system for dsRNA for RNAi pesticide action in agriculture.

Additionally, when dsRNA for pest control is sprayed on agricultural crops, improvement of dsRNA stability in ambient conditions is required. To reduce damage to naked RNA molecules by exogenous ribonucleases (RNases) or ultraviolet rays, some simple device to prevent the degradation as much as possible is necessary. Mitter et al. (2017) reported improvement of dsRNA stability with a special newly developed fine clay nanosheet. By employing the nanosheet as a dsRNA carrier, they achieved prolonged stability of dsRNA sprayed onto leaves and effective RNAi action. There is also a known method using heat-sterilized *E. coli* cells in which a target dsRNA was accumulated and then sprayed on fields without extracting the dsRNA from the microbial cells (Zhu et al. 2011). The method of directly using microbes containing a target dsRNA could reduce the cost of preparing the dsRNA for agricultural application.

In this study, as another method of providing stabilized dsRNA for practical use, we attempted to simply treat dsRNA-producing *C. glutamicum* cells with ethanol or methanol. It was found that stabilization of the dsRNA inside the cells occurred simultaneously with the alcohol treatment that resulted in bacterial killing. Thus, we here report an inexpensive preparation method for dsRNA that can be applied in agricultural uses in the future.

## Materials and methods

### Bacterial strains, plasmids, DNA primers, and media

Bacterial strains and plasmids used in this study are listed in Table 1. *C. glutamicum* 2256LΔ*rnc*, which is a strain deficient in RNase III (the *rnc* gene product, mainly involved in dsRNA degradation), was used as the host strain for dsRNA production. *C. glutamicum* strains were routinely grown on CM-Dex medium (Chinen et al. 2007) in a test tube at 30 °C and also cultured in a jar fermentor using RPB1 medium (Hashiro et al. 2019b). *Escherichia coli* JM109 (Takara Bio, Shiga, Japan) was used for construction of recombinant plasmids and cultured in Luria-Bertani medium at 37 °C. When appropriate, antibiotics were added as follows: chloramphenicol (Cm) at 5 mg/L and 20 mg/L for *C. glutamicum* and *E. coli*, respectively, and ampicillin (Ap) at 100 mg/L for *E. coli*. Plasmid pVC7H2, which is a high copy number mutant plasmid derived from *C. glutamicum*/*E. coli* shuttle vector pVC7N, was used as the vector for dsRNA expression. Plasmid pGEM-RD2 was used for evaluation of plasmid copy number in *C. glutamicum*. All DNA primers used in this study are listed in Supplemental Table S1 and were obtained from Eurofins Genomics (Tokyo, Japan).

**Table 1:** Bacterial strains and plasmids used in this study

Strain or plasmid	Relevant characteristic(s)	Reference
Strain		
*Corynebacterium glutamicum*		
2256	ATCC 13869, AJ1151, wild-type strain	Nishio et al. (2017)
2256L	2256 derivative cured of cryptic plasmid pAM330N	Hashiro et al. (2019a)
2256LΔ*rnc*	*rnc* mutant of 2256L	Hashiro et al. (2019b)
*Escherichia coli*		
JM109	*endA1*, *recA1*, *gyrA96*, *thi*, *hsdR17* (r_k_^−^, m_k_^+^), *relA1*, *supE44*, λ^−^, Δ *(lac-proAB*), F′[*traD36*, *proAB*^+^, *lacI*^q^, *lacZ*ΔM15]	Takara-Bio (Shiga, Japan)
Plasmid		
pAM330N	Cryptic plasmid in *C. glutamicum* 2256	Hashiro et al. (2019a)
pVC7N	*C. glutamicum*–*E. coli* shuttle vector derived from pAM330N and pHSG329; Cm^r^	Hashiro et al. (2019a)
pVC-U1A*-1	pVC7N derivative carrying F1 promoter derived from BFK20; Cm^r^	Hashiro et al. (2019b)
pVC7H2	pVC7N *copA2* mutant; Cm^r^	Hashiro et al. (2019a)
pVC-Pf1-HvIap	pVC7N derivative carrying part of the *diap1*-cDNA and F1 promoter; Cm^r^	This study
pVH2-Pf1rev	pVC7H2 derivative carrying F1 promoter derived from BFK20; Cm^r^	This study
pVH2-KX-Pf1rev	pVH2-Pf1rev derivative carrying *Xho*I and *Kpn*I sites downstream of F1 promoter; Cm^r^	This study
pVH2-HvIap-1	pVC7H2 derivative carrying *diap1*-dsRNA expression unit; Cm^r^	This study
pGEM-T Easy	Cloning vector, Ap^r^	Promega (Tokyo, Japan)
pGEM-RD2	pGEM-T Easy derivative containing *repA* gene fragment from pAM330N and *dnaA* gene fragment from *C. glutamicum* 2256L; Ap^r^	Hashiro et al. (2019a)

*Cm*^*r*^ resistance to chloramphenicol, *Ap*^*r*^ resistance to ampicillin

### Insect

*H. vigintioctopunctata* adults were collected at the National Institute for Basic Biology (Aichi, Japan) or Kumamoto University (Kumamoto, Japan). We established a line in the laboratory that was used for all experiments on the effects of dsRNA on the pest. They were reared on potato leaves at 25 °C. Larvae used in this study were derived from a few batches of eggs and were starved after second molting.

### Plasmid construction

**Fig. 1:**
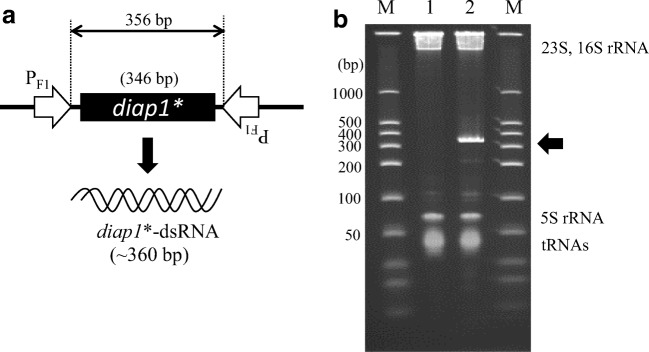
Expression of diap1*-dsRNA by *Corynebacterium glutamicum*. **a** Structure of diap1*-dsRNA expression system in pVH2-HvIap-1. Two F1 promoters (P_F1_) are indicated by open arrows facing each other. diap1* represents part of the *diap1*-cDNA region. **b** PAGE-analysis of produced diap1*-dsRNA. Lane M shows size markers of double-stranded RNAs. Lanes 1 and 2 are total RNA fractions from *C. glutamicum* strains 2256LΔ*rnc* harboring pVC7H2 as a control and pVH2-HvIap-1, respectively. A prominent RNA band corresponding to diap1*-dsRNA is indicated with an arrow, and intrinsic RNA species (23S rRNA, 16S rRNA, 5S rRNA, and tRNAs) from host cells are also indicated

RNA expression plasmid pVH2-HvIap-1 was constructed as follows. A DNA fragment (about 110 bp) containing the F1 promoter region was amplified by PCR using pVC-U1A*-1 (Table 1) as the template and primers P01 and P02. Next, the target diap1*-DNA fragment, part of the *diap1* gene of *H. vigintioctopunctata* (DDBJ acc. no. LC473084) (Supplemental Fig. S1) (Niimi et al. 2015), was obtained by PCR using a *diap1*-cDNA fragment as the template and primers P03 and P04. Using pVC7N as the template, a vector DNA fragment was obtained by PCR using primers P05 and P06. Then, the above three DNA fragments were linked by In-Fusion Ligation (Takara Bio) to construct pVC-Pf1-HvIap. Another DNA fragment containing the F1 promoter region was amplified by PCR using pVC-U1A*-1 as the template and primers P07 and P08. This DNA fragment was ligated with a vector fragment amplified by PCR with primers P05 and P11 using pVC7H2 as the template to construct pVH2-Pf1rev. This plasmid was subjected to inverse PCR amplification with primers P05 and P09; then, the amplified DNA fragment was treated with *Dpn*I and phosphorylated at the 5′- ends to construct pVH2-KX-Pf1rev by self-ligation of the processed DNA fragment. Next, using pVC-Pf1-HvIap as the template, PCR amplification was performed with primers P10 and P12 to prepare a DNA fragment containing the F1 promoter and diap1*-DNA region. Finally, this DNA fragment and pVH2-KX-Pf1rev were treated with restriction enzymes *Xho*I and *Kpn*I, and the DNA fragments were ligated with T4 ligase (Toyobo, Osaka, Japan) to construct plasmid pVH2-HvIap-1 (Fig. 1a). Thus, an expression system for diap1*-dsRNA was constructed in which two F1 promoters faced each other across the partial *diap1* coding fragment. The polymerase used for PCR in this study was PrimeSTAR GXL DNA polymerase (Takara Bio). The constructed plasmid was transformed into *C. glutamicum* strain 2256Δ*rnc* by electroporation.

### Production of dsRNA and PAGE analysis

For small-scale cultivation using test tubes, transformants were cultured in 2 mL of CM-Dex medium containing Cm at 30 °C for 18 h, and total RNA was prepared from 0.2 mL of the culture broth as described previously (Hashiro et al. 2019b). Total RNA solution (1 μL) was mixed with 7 μL of 150 mM NaCl and 2 μL of Hi-Density TBE sample buffer (Thermo Fisher, Tokyo, Japan), and then, the sample was analyzed by 6% nondenaturing PAGE (Thermo Fisher), followed by staining with SYBR Green II Nucleic Acid Gel Stain (Takara Bio). Batch fermentation using a BSS-01NP fermentor (ABLE Co., Tokyo, Japan) was performed in 0.3 L of RPB1 medium containing 40 g/L l-glucose at 30 °C, as described previously (Hashiro et al. 2019b). Cell growth was monitored by measuring the optical density of the culture at 620 nm (OD_620_) after 101-fold dilution of the culture with 0.2 M HCl. Quantification of produced RNA was carried out by PAGE analysis using DynaMarker dsRNA (BioDynamics Lab., Tokyo, Japan) as a standard for the calculation (Hashiro et al. 2019b). The copy number of plasmid and the ratio of segregates in culture broth after fermentation were determined as described previously (Hashiro and Yasueda 2018; Hashiro et al. 2019a).

### RT-PCR

One-step RT-PCR was performed with r*Tth* DNA polymerase according to the manufacturer’s protocol (Toyobo) to indicate the presence of an RNA region specific for the *diap1* gene. The sample RNA as template for reverse transcription was purified from a PAGE gel on which total RNA from *C. glutamicum* 2256LΔ*rnc*/pVH2-HvIap-1 was electrophoresed. After the purified RNA sample (2 μg) was treated with RNase-free DNase I (Qiagen, Tokyo, Japan) and extracted with TRIzol reagent (Thermo Fisher), the RNA (at concentrations in a serial tenfold dilution from 0.001 to 10 ng, as shown in Supplemental Fig. S2c) was denatured at 90 °C for 30 s, followed by reverse transcription at 60 °C for 30 min in 50 μL of reaction buffer containing 10 pmol of each primer (P13 and P14), 2.5 mM manganese (II) acetate, and 5 U r*Tth* DNA polymerase. After the denaturation, the generated cDNA was then amplified by 30 cycles of denaturation at 94 °C for 30 s, annealing at 60 °C for 30 s and extension at 72 °C for 30 s, following by incubation at 72 °C for 7 min. The products were electrophoresed on a 2% agarose gel with DNA size marker (1 kb Plus DNA ladder, Thermo Fisher).

### RNase digestion

RNase digestion experiments using RNase III (Thermo Fisher) and RNase A (Promega, Tokyo, Japan) were carried out to characterize the structural features of the produced recombinant RNA. Total RNA (1.6 μg) extracted from dsRNA-producing cells was digested at 25 °C for 30 min in 15 μL reaction solution with 1000 U of RNase A. In RNase III digestion, the RNA sample was reacted with RNase III (500 U) at 37 °C for 2 h in 16 μL reaction solution containing 10 mM Tris-HCl (pH 7.5), 10 mM EDTA, and 100 mM NaCl. Both digestion reactions were loaded on a 6% acrylamide gel after addition of loading buffer (Hi-Density TBE sample buffer).

### Treatment of *C. glutamicum* cells with alcohol

*C. glutamicum* 2256LΔ*rnc*/pVH2-HvIap-1 and the control strain *C. glutamicum* 2256LΔ*rnc*/pVC7H2 were cultured in test tubes as described above. After 0.2 mL of the culture broth were centrifuged (13,800×*g*, 2 min) to collect the cells, 1 mL of 10 mM phosphate buffer solution (pH 6.8) or buffer solution containing each concentration of alcohol (ethanol or methanol, from 10 to 95% [v/v], as indicated in Fig. 4) was added to the cell pellet at 20 °C, and then the cells were thoroughly resuspended. The cell concentration during the alcohol treatment was about 10^9^ cells/mL in the reaction tube, and the suspension was allowed to stand at 20 °C for 10 min. After the treatment, the cell suspension was centrifuged to remove the supernatant. To examine the survival rate of treated cells, 0.2 mL of CM-Dex medium was immediately added to the cell pellet and then 0.1 mL of the suspension was spread and cultured on CM-Dex agar medium at 30 °C for 48 h.

Evaluation of dsRNA stability in the alcohol-treated cells was performed as follows: each cell pellet treated with alcohol was incubated at 20 °C for an additional 24 h. After the incubation, the total RNA was extracted from the incubated cells and analyzed by 6% PAGE for detection of the target dsRNA band. When preparing the large amount of dsRNA-containing cells necessary for feeding to insect larvae, such sterilized microbial cells were prepared from 1.5 mL of culture medium.

### Heat treatment of *C. glutamicum* cells

*C. glutamicum* cells containing target dsRNA and the control strain were sterilized by heating as follows: 0.2 mL of the culture medium was placed into a microtube and centrifuged in the same manner as described above. The cells were washed with 1 mL of 10 mM phosphate buffer solution (pH 6.8) and then centrifuged again at 4 °C to obtain the microbial cells. The sample was placed into a heat block (Dry Thermounit DTU-1B; TAITEC Co., Saitama, Japan) at 100 °C and incubated for 30 min for sterilization. After cooling to room temperature, 0.2 mL of CM-Dex medium was added to the heat-treated cells and they were resuspended; 0.1 mL of the suspension was spread on CM-Dex agar medium, and the viability of the cells was examined. The stability of dsRNA in the producing cells was evaluated by preparing total RNA from the cells after standing at 20 °C for an additional 24 h after the heat treatment and examining it by PAGE.

### Feeding of microbial cells and evaluation of vital activities of *H. vigintioctopunctata*

Sterilized *C. glutamicum* cells (60 mg wet weight) were rinsed with 1 mL of water twice and suspended in 50 μL of sterilized water; 0.5 μL of the suspension was spotted on a siliconized glass slide (76 × 26 mm) and fed to an early third instar larva of *H. vigintioctopunctata* that had been starved after the second molt. After feeding, the larva was transferred onto a potato leaf and raised for 24 h. After that, the larva was transferred to new potato leaf and the rearing was continued for another 24 h. As evaluation indexes of the effect of administration of the dsRNA, the area of leaf eaten was measured in the periods 0–24 and 24–48 h after feeding the *C. glutamicum* cells, and the body weight of the larvae was examined at 48 h. To measure the feeding area of leaf (i.e., the area that had been eaten), a digital microscope system (VHX-5000, KEYENCE, Osaka, Japan) was used, and an analytical balance (AUW120D, Shimadzu, Kyoto, Japan) was employed for body weight measurement. We performed the Welch’s *t* test to verify the differences of effects of *diap1* RNAi assay. The Holm’s method was applied in the multiple comparisons. The significance level in our analysis was *P* value < 0.05. All statistical analyses were performed by R-3.4.2 (https://cran.r-project.org/).

### qPCR

Total RNA was extracted from the whole body of *H. vigintioctopunctata* using TRI Reagent (Molecular Research Center, Cincinnati, OH, USA), and DNase I treatment (1 units; New England Biolabs, Beverly, MA) was performed at 37 °C for 30 min in order to thoroughly degrade genomic DNA that might be contaminated in the RNA solution. First-strand cDNA was synthesized from 1 μg of the total RNA using SuperScript III Reverse Transcriptase (Life Technologies, Tokyo, Japan) and primer P19. qPCR was performed using THUNDERBIRD SYBR qPCR Mix (Toyobo) according to the manufacturer’s protocol in a LightCycler 96 instrument (Roche, Basel, Switzerland). The expression level of *diap1* relative to the internal control, *ribosomal protein 49* gene (*rp49*; accession number: AB480201), was calculated by the 2^−ΔΔ*C*t^ method (Livak and Schmittgen 2001); primer pairs P17 and P18, and P15 and P16, were used respectively (nucleotides 1649–1783 of *diap1*-cDNA [Supplemental Fig. S1]; Chikami et al. 2019). We performed Welch’s *t* test to evaluate the qPCR analysis using R-3.4.2. The *P* values were adjusted by Holm’s method in the multiple comparison, and we adopted *P* < 0.05 as the significance level.

## Results

### Construction of diap1*-dsRNA overproduction system

The *diap1* gene in *H. vigintioctopunctata* was selected as a target gene for RNAi action in the pest, and a 346-bp region of *diap1*-cDNA (DDBJ acc. no. LC473084, nucleotides 886–1231 in Supplemental Fig. S1) was employed for production of diap1*-dsRNA. For the dsRNA overexpression, we adapted a convergent transcription system using a pair of strong F1 promoters from corynephage BFK20. The expression unit was cloned into a high-copy number vector, pVC7H2, to yield plasmid pVH2-HvIap-1 for the diap1*-dsRNA overexpression (Fig. 1a). After the plasmid was introduced into *C. glutamicum* 2256LΔ*rnc*, the transformant was cultured in a test tube and the total RNA was extracted for investigation of production of the target dsRNA. *C. glutamicum* 2256LΔ*rnc* harboring pVH2-HvIap-1 produced a target RNA species having the expected length (about 360 bp) (Fig. 1b).

**Fig. 2:**
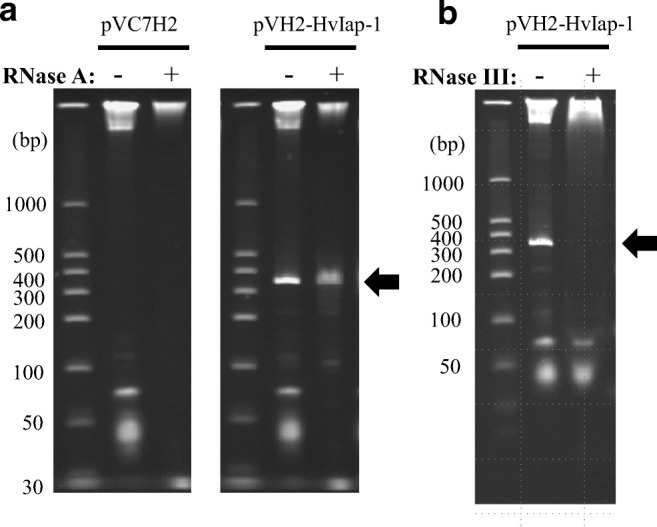
Identification of diap1*-dsRNA produced in *C. glutamicum*. Structural analysis of the produced RNA with RNase A (**a**) and RNase III (**b**). Total RNA from *C. glutamicum* strain 2256LΔ*rnc* harboring pVC7H2 or pVH2-HvIap-1 was prepared, and then, each RNA sample treated with RNase (plus sign) or not treated (minus sign) was subjected to 6% PAGE. Prominent RNA bands corresponding to diap1*-dsRNA are indicated with black arrows. Size marker of dsRNAs is also shown

Then, we examined the structural features of the produced RNA by processing reactions with RNase A and RNase III. RNase A is an endoribonuclease that specifically degrades ssRNA at pyrimidine nucleotides, while RNase III is a dsRNA-specific endonuclease. The results indicated that the RNA species produced by this system had a double-stranded structure because the RNA was decomposed by RNase III but not by RNase A (Fig. 2). In *C. glutamicum* 2256L (an *rnc*^+^ strain) harboring pVH2-HvIap-1, PAGE analysis of the total RNA fraction did not detect an RNA species of the expected size but instead showed products that appeared to be degraded (Supplemental Fig. S3); this also suggested that the RNA species produced in the *rnc*^*−*^ strain (*C. glutamicum* 2256LΔ*rnc*) had a double-stranded structure. In addition, RT-PCR using a pair of primers specific to the target coding region of *diap1*-cDNA (Supplemental Fig. S2b) showed the produced RNA chain had the nucleotide sequence of the region targeting the *diap1* gene (Supplemental Fig. S2), judging from the result that the size of the PCR product was consistent with the DNA length expected to be amplified by the primers used. Therefore, we concluded that the RNA produced was a dsRNA containing the desired mRNA region from the target gene *diap1*. Taken together, these data show that the constructed expression system can efficiently produce diap1*-dsRNA.

### Production of diap1*-dsRNA in *C. glutamicum* in a fermentor

**Fig. 3:**
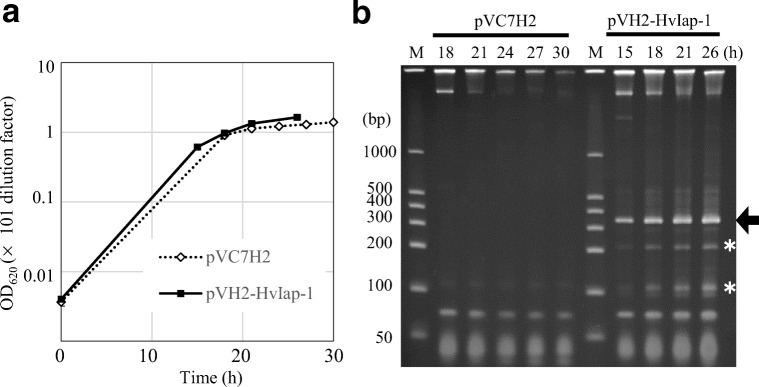
Production of diap1*-dsRNA by *C. glutamicum* in batch fermentation. **a** Growth of *C. glutamicum* 2256LΔ*rnc* harboring pVC7H2 as a control or pVH2-HvIap-1. Average values and standard deviations (SDs) for three independent experiments are shown, although the variation range is too small to distinguish the SDs on the graph. **b** PAGE analysis of total RNA prepared from each *C. glutamicum* strain during the fermentation. Lane M shows dsRNA marker, and each culture time (h) is indicated at the top of the gel. The arrow and asterisks indicate the positions of diap1*-dsRNA and its possible degradation products, respectively. The result presents one representative diap1*-dsRNA production experiment in a jar fermentor

In response to large sample preparations for future experiments using *H. vigintioctopunctata*, we performed batch culture of the diap1*-dsRNA-producing strain in a jar fermentor. Typical growth curves of the diap1*-dsRNA producing strains and the control strains harboring vector pVC7H2 are shown in Fig. 3a, each using three transformants. Although the cell density of all strains reached OD_620_ ≈ 100 as the maximum value, the RNA-producing strains and the control strains consumed 40 g of l-glucose by 26 and 30 h of fermentation, respectively. The total RNA was extracted from cultured cells at several time points during the cultivation and the RNA content was analyzed by PAGE. A representative result is shown in Fig. 3b. The target diap1*-dsRNA accumulated as a major and discrete RNA species as the culture progressed. In the latter period of the fermentation, the presence of RNA species that appeared to be degradation products of diap1*-dsRNA was observed at a low level. In addition, some products at higher molecular weight than the original diap1*-dsRNA band were faintly detectable by PAGE. However, it was clear that the dsRNA-expressing microbes could efficiently accumulate the desired diap1*-dsRNA within the cells. We obtained a maximum yield of about 75 mg per liter of culture of the diap1*-dsRNA in this fermentation, and we could prepare *C. glutamicum* accumulating the target diap1*-dsRNA within cells.

The stability of the plasmids maintained by the cells was examined. In the control strains, almost no segregates were observed even in the last stage of the culture, and the copy number of the vector plasmid was maintained at about 300 as originally determined. However, in the culture of the diap1*-dsRNA expressing strains, the fraction of cells harboring pVH2-HvIap-1 was reduced to around half, suggesting some maintenance instability of the RNA expression plasmid in this strain.

### Treatment of *C. glutamicum* with alcohol or heating

As a first step in the preparation of sterilized microbes containing dsRNA, we examined the ethanol concentration required to sterilize *C. glutamicum* cells producing diap1*-dsRNA. Generally, ethanol concentration of around 80% is most effective for the sterilization of microorganisms (Suchomel and Rotter 2011; McDonnell and Russell 1999); thus, we investigated whether such conditions could also be applied to the sterilization of *C. glutamicum* accumulating the dsRNA in this study. The treatment conditions were tentatively set such that the bacterial concentration was about 10^9^ cells/mL, and the treatment temperature and duration were 20 °C and 10 min, respectively. The survival of *C. glutamicum* cells was found to be very low at ≥ 30% (v/v) ethanol (Supplemental Table S2); not one microbial colony appeared on the detection agar-medium plates, indicating that the survival rate of the cells was at most 10^−8^.

**Fig. 4:**
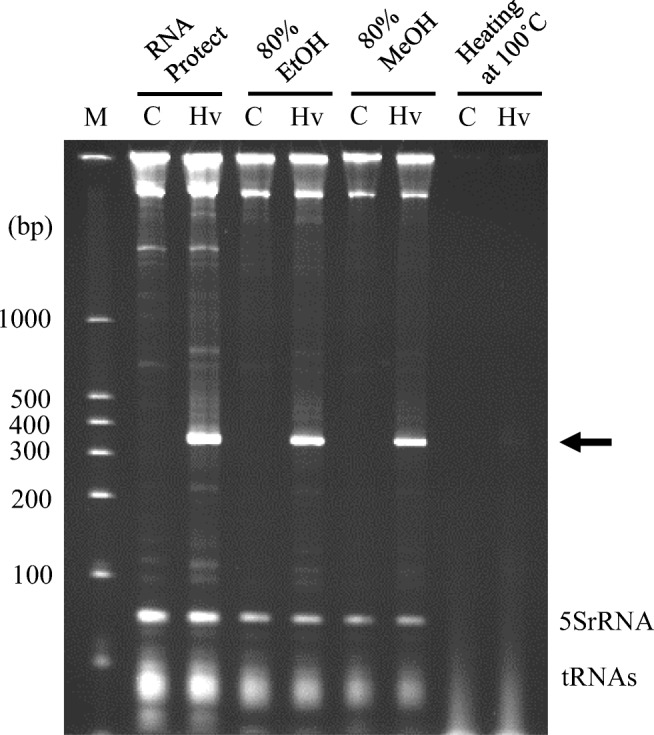
Analysis of RNAs extracted from *C. glutamicum* cells treated with alcohol or by heating. Lanes C and Hv indicate total RNA fractions from *C. glutamicum* cells harboring pVC7H2 as a control and pVH2-HvIap1, respectively. Lane M shows RNA size markers. “RNA protect” indicates RNA fractions extracted from microbial cells treated immediately with RNA Protect Bacteria Reagent without alcohol or heating treatment. Each treatment method applied for the sterilization is indicated at the top of the gel. RNA samples were analyzed on nondenaturing 6% PAGE, and the position of diap1*-dsRNA is indicated by a black arrow

Next, the stability of the dsRNA within the sterilized cells was examined. *C. glutamicum* cells were separately treated with ethanol at concentrations from 30 to 95% (v/v). Each collected cell pellet was incubated at 20 °C for an additional 24 h, and then, total RNA was extracted from the individual tubes and subjected to PAGE to examine the integrity of the target dsRNA. Decomposition of the dsRNA hardly occurred when the ethanol concentration was ≥ 50% (v/v) (Supplemental Fig. S4). When dsRNA-producing cells not treated with ethanol were left to incubate in a similar way (as a negative control), or when the ethanol concentration was ≤ 30% (v/v), significant RNA degradation was observed. Therefore, considering the stabilization of the dsRNA in the sterilized microbe as an index of effectiveness, we chose 80% (v/v) for ethanol treatment of the dsRNA-producing strain of *C. glutamicum* (Fig. 4).

In a similar way as for ethanol, the survival of *C. glutamicum* cells in methanol and its effect on dsRNA accumulation in the cells were investigated. Sterilization of the microbes and intactness of the dsRNA were observed when the methanol concentration was ≥ 70% (v/v) (Supplemental Fig. S5); 80% (v/v) methanol was chosen for treatment of dsRNA-producing *C. glutamicum* cells (Fig. 4), according to the concentration used for ethanol treatment.

Sterilization of the cells by heating was also assessed. It was necessary to treat *C. glutamicum* cells at 100 °C for at least 30 min for complete killing of the microbes in a microreaction tube in a heat block. Heat killing of the cells resulted in severe degradation of RNAs, including the target diap1*-dsRNA (Fig. 4).

### Effect of ingestion of sterilized cells containing diap1*-dsRNA on vital activity of the pest *H. vigintioctopunctata*

**Fig. 5:**
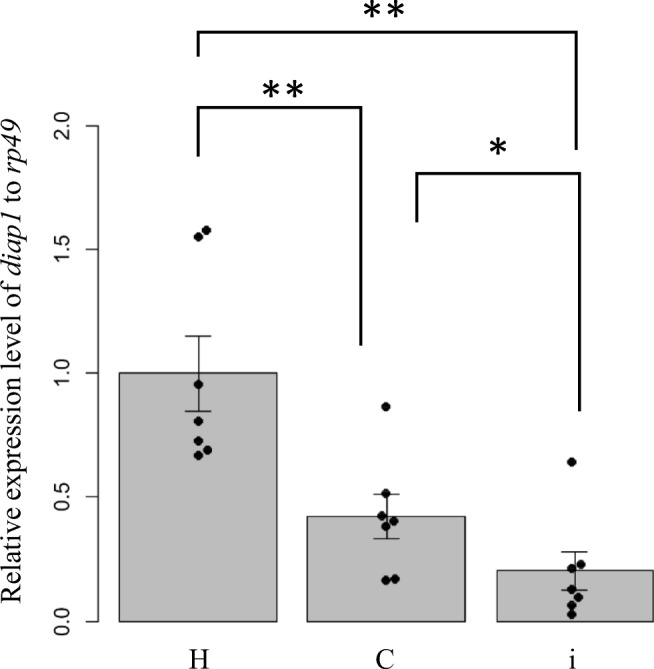
Evaluation of *diap1* gene expression in *H. vigintioctopunctata* by qPCR. The relative gene expression level of *diap1* to *rp49* as a control was examined. The samples were prepared from whole bodies of larvae that ingested only water (H), ethanol-sterilized cells harboring pVC7H2 vector (C), and ethanol-sterilized cells harboring pVH2-HvIap-1 (i), respectively. Statistical significance was calculated using Welch’s *t* test. The data represent the mean ± standard error (SE) (*n* = 7). *P < 0.05, **P < 0.01

To examine the effect of diap1*-dsRNA on *H. vigintioctopunctata*, ethanol-sterilized *C. glutamicum* cells containing the dsRNA were suspended in a small amount of water and fed to third instar larvae of the pest. In the control groups, only water or sterilized cells containing empty vector (pVC7H2) was fed to the pest. First, the expression level in each pest of the *diap1* gene targeted by the RNAi action was assayed by qPCR. Total RNA was prepared from the whole body using larval individuals reared for 48 h after ingestion of each sample (water only, control sterilized cells, or sterilized cells containing the dsRNA) and then subjected to qPCR. *diap1* expression in *H. vigintioctopunctata* in the group of larvae fed the control sterilized cells was significantly reduced by approx. 59%, compared with those fed water. In the larvae fed sterilized cells containing diap1*-dsRNA, the *diap1* expression level was further decreased to approx. 21% of that in those fed water (Fig. 5). These results indicated that intake of sterilized *C. glutamicum* cells itself caused physiological changes associated with reduction of *diap1* expression in the pest. It was also demonstrated that the presence of diap1*-dsRNA could further suppress *diap1* expression in the larvae.

**Fig. 6:**
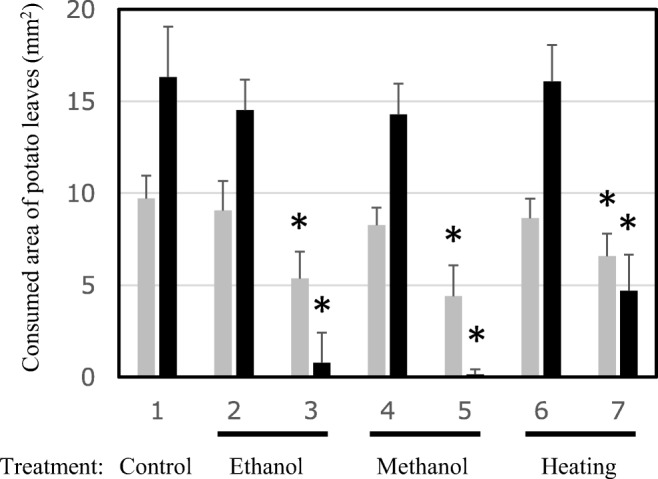
Effect of feeding H. vigintioctopunctata diap1*-dsRNA-producing *C. glutamicum* cells treated with alcohol or by heating on leaf consumption activity*.* After feeding the sample, the areas of leaf consumed during hours 0–24 (gray bars) and subsequently during hours 24–48 (black bars) were measured. Sample 1, larvae fed water as a control; 2, 4, 6, larvae fed *C. glutamicum* 2256LΔ*rnc* cells harboring pVC7H2 vector; 3, 5, 7, larvae fed *C. glutamicum* 2256LΔ*rnc* cells harboring pVH2-HvIap-1. The data are presented as the mean ± SD. For each group, 4–5 larvae were used. Asterisks indicate significant differences (*P* < 0.05) from corresponding values for the strain harboring only vector

**Fig. 7:**
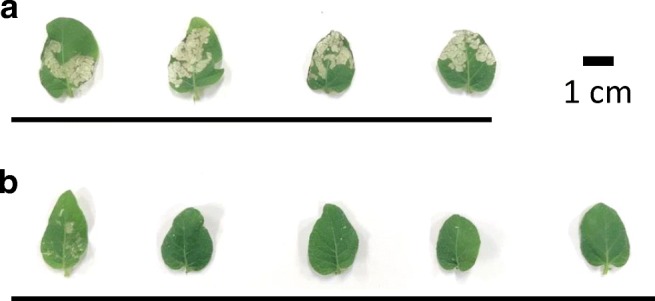
Damage to potato leaves by larvae of *H. vigintioctopunctata* and its suppression by feeding sterilized microbial cells containing diap1*-dsRNA to the pest. *C. glutamicum* cells harboring pVC7H2 vector (**a**) or pVH2-HvIap1 (**b**) were treated with 80% ethanol for sterilization and then fed to *H. vigintioctopunctata* larvae. The status of potato leaves eaten between 24 and 48 h after the feeding of sterilized cells to the larvae is shown. A typical result from four to five leaves from each experimental group is presented. Scale bar, 1 cm

Next, changes in eating of potato leaves by treated *H. vigintioctopunctata* larvae were examined. Larvae in the control group, which ingested only water, consumed about 10 mm^2^ after rearing for 24 h, and about 16 mm^2^ during 24–48 h after water intake (Fig. 6). *C. glutamicum* cells carrying only the vector pVC7H2 were treated by each of the three sterilization methods (ethanol, methanol, or heating), and these were given to the pest larvae. When compared with the control (water) group, no significant difference in the amount of leaf eating was observed (Fig. 6; Supplemental Table S3). However, in the case of larvae fed sterilized cells containing diap1*-dsRNA, a significant reduction in the eating of potato leaves was observed regardless of the *C. glutamicum* cell killing method (Fig. 6; Supplemental Table S4). It was also shown that the feeding activity of the pests was reduced more by the intake of cells prepared by sterilization with alcohol than with cells killed by heating. In particular, feeding activity of larvae was hardly observed after 24 h after intake of cells sterilized with alcohol (Figs. 6 and 7).

**Fig. 8:**
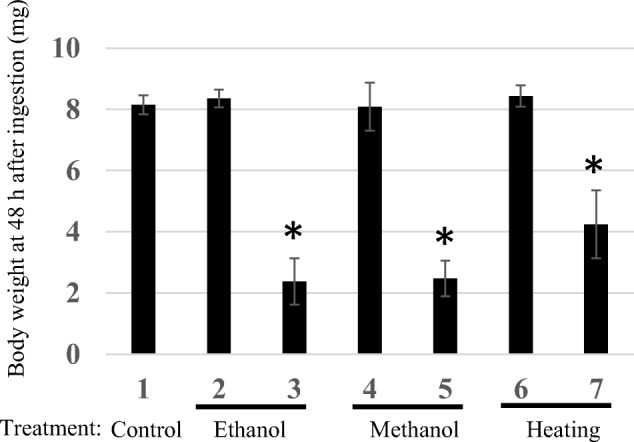
Evaluation of larval body weight of *H. vigintioctopunctata* 48 h after ingestion of *C. glutamicum* cells sterilized by alcohol or heating*.* Sample 1, larvae fed water (negative control); 2, 4, 6, larvae fed *C. glutamicum* 2256LΔ*rnc* cells harboring pVC7H2 vector; 3, 5, 7, larvae fed *C. glutamicum* 2256LΔ*rnc* cells harboring pVH2-HvIap-1. The data are presented as the mean ± SD. For each group, 4–5 larvae were used. Asterisks indicate significant differences (*P* < 0.05) from corresponding values for the strain harboring only vector

Furthermore, the effect of intake of sterilized *C. glutamicum* cells containing diap1*-dsRNA on the growth of larva was evaluated using changes in the body weight as an index. In controls, water only was given to the third instar larva. The average weight after rearing them on potato leaves for 48 h was about 8 mg (Fig. 8). The weight value was almost the same in the group fed cells containing the vector only. When sterilized cells containing diap1*-dsRNA were fed, the average weights were significantly lower, regardless of the *C. glutamicum* sterilization method, and only slight growth was observed from the larvae of the start stage in the experiment (Supplemental Table S4). The body weight of the larvae was lower when fed microbial cells sterilized with alcohol than heat-killed cells (Fig. 8).

Taken together, these results indicated that *C. glutamicum* cells containing diap1*-dsRNA sterilized with alcohol were able to significantly suppress the vital activity of *H. vigintioctopunctata* larvae. In the case of this pest at least, alcohol-treated *C. glutamicum* cells could be used as capsules containing target dsRNA, which could be applied as a new biopesticide.

## Discussion

In previous study, we constructed an expression system that can produce large amounts of recombinant RNA using *C. glutamicum* as the host microbe (Hashiro et al. 2019b). Here, based on this overexpression system, we adopted a convergent transcription system for producing dsRNA to suppress *diap1* gene expression in a target insect, *H. vigintioctopunctata*; high productivity of recombinant dsRNA was obtained (around 75 mg/L) (Figs. 1 and 3). In the RNA expression system, no special transcriptional terminators were used for termination of strong transcription from the phage-derived promoter F1, but each complementary ssRNA transcribed from the target RNA-coding region on the expression plasmid was inferred to be paired immediately after transcription and trimmed by some kind of RNase in vivo. Thus, the target dsRNA appeared to be efficiently accumulated in this convergent expression system in *C. glutamicum* (Fig. 3). Several studies on dsRNA production by a convergent transcription system using T7-RNA polymerase/the T7 promoter in *E. coli* strain HT115 (*rnc*^*−*^) have been reported (Wan et al. 2015; Zhu et al. 2011; Ratzka et al. 2013), but the production levels of target dsRNAs remain obscure, although Tenllado et al. (2003) stated that their target dsRNA production using the *E. coli*/T7 system was about 4 mg/L of culture medium. In many studies on pest growth control using dsRNA, the various target dsRNA species have been prepared using in vitro transcription systems. However, such an approach is obviously expensive and not suitable for the production of large amounts of target dsRNAs. In that respect, our microbial dsRNA production system could be applicable for large-scale dsRNA production for field testing of the dsRNA. *C. glutamicum* is a nonpathogenic, “Generally Recognized as Safe” microbe, used globally for fermentative production of huge amounts of amino acids (Lee and Wendisch 2017; Ikeda and Takeno 2013; Yasueda 2014), indicating that this microbe is robust. It seems that *C. glutamicum* is also very suitable for mass production of recombinant RNA molecules at low cost.

We showed inhibition of the growth of *H. vigintioctopunctata* larvae by direct ingestion of dsRNA for RNAi of the *diap1* gene in previous work (Niimi et al. 2015; Chikami et al. 2019). Here, it has been demonstrated that the vital activity of this pest can be suppressed even by feeding it sterilized microbial cells containing diap1*-dsRNA. It is known that the growth of Colorado potato beetle (CPB), *Leptinotarsa decemlineata*, a famous crop pest, is also affected adversely by suppression of target gene expression due to feeding of dsRNA (Sun Miguel and Scott 2016; Wan et al. 2015). In particular, Zhu et al. (2011) treated *E. coli* cells which produced a target dsRNA for suppressing the growth of CPB with heating to sterilize the microbes, and then the treated cells were sprayed onto foliage to show their effectiveness as a pesticide. The method is an effective means to provide target dsRNA for pest control at low cost. However, in heating treatment for sterilization, if the volume of the object to be heated is large, the thermal conductivity to the whole object is somewhat slow, and thus, it might be difficult to ensure uniformity of heating of the whole of the reactant to be processed. Therefore, to completely sterilize the objective microbial cells by heating, it is supposed that excessively heated cells are formed, in which the target RNA can be decomposed. Our experiments with *C. glutamicum* cells support this conclusion as we observed substantial RNA degradation during heat treatment (Fig. 4), although the result might have been specific to the heat-sterilizing conditions or host strain used in this study. As an alternative simple and efficient sterilization method, we studied sterilization of *C. glutamicum* while maintaining the integrity of dsRNA accumulated inside the cells by using ethanol or methanol. In particular, ethanol is a disinfectant widely used in food hygiene, and the use of ethanol in the preparation of such an RNA insecticide will have relatively little environmental burden. These alcohols rapidly permeate into the inside of microbial cells while destroying part of the cell membrane structure, thereby causing denaturation of proteins in the microbe (McDonnell and Russell 1999). This could effectively suppress the activity of RNA-degrading enzymes remaining in the producing microbial cells. Thus, it seems that nucleic acids are partially dehydrated by alcohol and then are stabilized inside the microbial cell bodies. In addition, this treatment can be carried out at room temperature or below, therefore suppressing degradation of the RNA molecules during this treatment process.

However, the membrane structure of the microbial cells is still maintained to some extent by this treatment process, and it is expected that the packaging of the target dsRNA by the treated cell envelope plays a role in protecting the internal dsRNA against, for example, exogenous RNases and ultraviolet rays. The ladybird beetles that ingested sterilized *C. glutamicum* cells containing diap1*-dsRNA no longer ate potato leaves voraciously, and their weight gain was suppressed (Figs. 6, 7, and 8). As these microbial cells can be used as capsules enclosing the target dsRNA, their insecticidal effect after spraying in agricultural fields should be evaluated. In heat-treated *C. glutamicum* cells, the RNA species were considerably degraded. However, feeding larvae these heat-processed cells containing diap1*-dsRNA still inhibited larval growth to some extent (Figs. 6 and 8). Since the RNA chain length actually required for RNAi action is about 21–23 bp, it is possible that some of the dsRNA fragments degraded by this heat treatment retained the length required for RNAi action.

In the case of the *diap1* gene, the phenotype that *H*. *vigintioctopunctata* feeding activity was suppressed appeared in a short period after ingestion of the dsRNA-containing *C. glutamicum* cells (Fig. 6), indicating that *diap1* is an excellent candidate gene as a target for an RNA-based pesticide (Niimi et al. 2015; Chikami et al. 2019). From the results of qPCR quantification of *diap1* expression in the pest, it was shown that the expression was significantly reduced even in larvae fed sterilized cells not containing diap1*-dsRNA (compared with the control larvae that only ingested water) (Fig. 5). However, no remarkable change was found in the leaf-feeding activity or weight gain of such larvae compared with water-ingesting control larvae (Figs. 6 and 8). Thus, the reduction in *diap1* expression in the larvae fed microbes that did not contain diap1*-dsRNA may be fluctuation of gene expression due to normal physiological responses to ingested bacteria or their fragments in the pest midgut. Although there is not yet sufficient knowledge about physiological actions, including immune responses, to exogenous bacteria in the midgut of ladybird beetles including *H*. *vigintioctopunctata*, there are some reports of responses to infectious bacteria in *Drosophila* gut (Buchon et al. 2009). In that insect, reactive oxygen species (ROS) are produced to eliminate bacteria that have invaded the gut, but the ROS also damage gut epithelial cells of the insect. Intestinal stem cells divide and proliferate rapidly to repair the damage to the tissue, and the appearance of apoptotic cells has been confirmed in the gut (Buchon et al. 2009). Therefore, in our present study, it is plausible that *diap1* expression in intestinal cells was physiologically suppressed by the signal of bacterial invasion, as a normal response. An immune response to infectious bacteria in the gut of *Drosophila* is induced by sensing peptidoglycan (PG), which constitutes the bacterial cell wall, through PG recognition proteins (Kurata 2014). *C. glutamicum*, which is a Gram-positive bacterium, is covered with a thick layer of mycolic acids (MA) on the outside of the cell but has a PG layer on the inside of the MA layer (Lanéelle et al. 2013). Therefore, it can be inferred that such an immune response may be triggered when the microbes enter the midgut of *H*. *vigintioctopunctata* even if the microbes are not alive. It is important to note, however, our data show that the reduction in *diap1* expression caused by the ingestion of diap1*-dsRNA is an enforced reduction in specific gene expression, not the normal physiological response of *H*. *vigintioctopunctata* to foreign bacterial bodies. Thus, *H*. *vigintioctopunctata* that ingest diap1*-dsRNA suffer marked inhibition of their biological activity. It has not been confirmed here that the target pest is killed by the intake of the diap1*-dsRNA; we speculate that this RNAi acts only in certain cells of the pest body and thus does not lead to rapid death of the pest (at least in the third instar larval stage, as used in our experiments). However, this dsRNA was shown to be able to exert the function of suppressing crop damage, which is crucial for a pesticide.

Since the dsRNA-producing *C. glutamicum* cells used in this study were sterilized until below the detection limit of live microbial cells, they would not be classified as living modified organisms (LMOs) based on the standard definition (Husby 2007). However, this RNA expression system carries a chloramphenicol resistance gene on a plasmid within the dead microbe. It will therefore be desirable to use a new selection marker in place of an antibiotic resistance gene marker, for example, the combination of a gene encoding an essential metabolic enzyme of the microbe on a plasmid and deficiency of that gene in the chromosome of the host microbial cell. In this fermentation test, it was found that the maintenance stability of the dsRNA expression plasmid in the production strain was not perfect. Indeed, in the culture of the dsRNA-producing strain, the time taken for complete consumption of glucose was shorter and the OD value of cells reached was slightly higher, compared with the control strain. This is probably the result of the emergence of segregates that have lost the expression plasmid. In the late stage of culture, the fact that the antibiotic concentration in the medium was below the action limit would be the cause of appearance of the segregates. Therefore, by using the plasmid maintenance approach described above, plasmid stability could be also improved, which will further increase production and accumulation of the target RNA.

RNAi acts specifically on a target gene sequence, and, therefore, RNA-based pesticides exert their action only on the target pest or an organism having the same sequence in a gene. The high specificity of target action suggests that the use of RNA-based pesticides is unlikely to result in side effects on beneficial insects such as honeybees. In addition, when resistance to a certain RNA-based pesticide appears in the target pest, newly effective ones could be produced rapidly, since it is sufficient to modify the base sequence of the dsRNA used. Taken together, this system for production of dsRNAs and their preparation is expected to contribute to a significant reduction in the cost of supplying dsRNAs that act on target agricultural pests, and we believe that the methods described in this paper provide an effective means of applying such dsRNAs as RNA-based pesticides in agriculture in the near future.

## Electronic supplementary material

ESM 1 (PDF 989 kb)
