# Supplementary material for: Construction of Corynebacterium glutamicum cells as containers encapsulating dsRNA overexpressed for agricultural pest control
Source: Appl Microbiol Biotechnol. 2019 Sep 5;103(20):8485–96. doi: 10.1007/s00253-019-10113-9 (PMC6800400; doi:10.1007/s00253-019-10113-9)
Supplement: Supplementary file 1 — (PDF 989 kb) [file 253_2019_10113_MOESM1_ESM.pdf]

Applied Microbiology and Biotechnology

Construction of *Corynebacterium glutamicum* cells as containers encapsulating dsRNA overexpressed for agricultural pest control

Shuhei Hashiro[1], Mayu Mitsuhashi[1], Yasuhiko Chikami[2,3], Haruka Kawaguchi[2,3], Teruyuki Niimi[2,3] and Hisashi Yasueda[1,4]*

[1] Institute for Innovation, Ajinomoto Co., Inc., 1-1 Suzuki-cho, Kawasaki-ku, Kawasaki 210-8681, Japan

[2] Division of Evolutionary Developmental Biology, National Institute for Basic Biology, Nishigonaka 38, Myodaiji, Okazaki, Aichi 444-8585, Japan

[3] Department of Basic Biology, School of Life Science, SOKENDAI (The Graduate University for Advanced Studies), Nishigonaka 38, Myodaiji, Okazaki, Aichi 444-8585, Japan

[4] Research and Development Center for Precision Medicine, University of Tsukuba, 1-2 Kasuga, Tsukuba-shi, Ibaraki 305-8550, Japan

*To whom correspondence should be addressed:
Hisashi YASUEDA, E-mail: hisashi_yasueda@ajinomoto.com

Supplementary materials

TABLE S1. Oligonucleotide DNA primers used in this study

Primer No. (name)	Primer sequence (5'-3')
P01 (F1P-20)	TAGGCACCCCAGGCTGATCTACTCGTTACTCAAGGC
P02 (F1P-21)	TCGCTATCTACTGTAACATACTC
P03 (HvI-22)	TACAGTAGATAGCGACCTCGGAATCGGCGACCAG
P04 (HvI-23)	TAGAAATACAACAGCCAATTC
P05 (VC-18)	AGCCTGGGGTGCCTAATGAG
P06 (VC-19)	GCTGTTGTATTTCTAAAGGCCAGGAACCGTAAAAG
P07 (F1P-25)	ACGGTTCCTGGCCTTGATCTACTCGTTACTCAAGGC
P08 (F1P-26)	TAGGCACCCCAGGCTTCGCTATCTACTGTAACATACTC
P09 (F1P-27)	GGTACCGGATCCCCTCGAGTCGCTATCTACTGTAACATACTC
P10 (F1P-28)	CCAGGTACCGATCTACTCGTTACTCAAGGC
P11 (VH2-24)	AAGGCCAGGAACCGTAAAAAG
P12 (IA1-29)	CGAACTCGAGTAGAAATACAACAGCCAATTC
P13 (rtPCR1)	CGCTTTGGTTCCCCCAGTG
P14 (rtPCR2)	TAGAAATACAACAGCCAATTC
P15 (rp49_f)	ATAGGGTTAGAAGACGTTTCAAGGG
P16 (rp49_r)	CTTCCAATTCTCTGACATTATGCAC
P17 (iap_f)	ATTGAATTTGGCAAGCATGAGGACA
P18 (iap_r)	CAGTAGTACGTCGAAGTGTCAGTGA
P19 (3'CDS)	AAGCAGTGGTATCAACGCAGAGTACTTTTTTTTTTTTTTTTTTTTTTTTTTTTVN

*Kpn*I and *Xho*I sites in the primer sequence are underlined and double underlined, respectively.

TABLE S2. Viability of *diap1-dsRNA producing *C. glutamicum* treated with alcohol**

	Viability[a] at the concentration [% (v/v)] of alcohol									
Alcohol	0	10	20	30	40	50	60	70	80	90
Ethanol	+++	+++	+++	$<10^{-8}$	$<10^{-8}$	$<10^{-8}$	$<10^{-8}$	$<10^{-8}$	$<10^{-8}$	$<10^{-8}$
Methanol	+++	+++	+++	+++	+	$<10^{-8}$	$<10^{-8}$	$<10^{-8}$	$<10^{-8}$	$<10^{-8}$

[a] +++, confluent or very heavy growth over whole plate; +, good growth of colonies over whole plate; $<10^{-8}$, no colony formed on plate.

TABLE S3. Statistical analysis on vital activities between larvae that ingested water and larvae that ingested cells containing the vector only

Treatment	Consumed leaf area						Body weight		
	0-24 hrs			24-48 hrs					
	t-value	*P*-value	Adjusted *P*-value	*t*-value	*P*-value	Adjusted *P*-value	*t*-value	*P*-value	Adjusted *P*-value
EtOH	0.6381	0.5484	0.5484	1.1193	0.3148	0.6296	-0.96202	0.3734	0.7468
MeOH	1.8448	0.1176	0.3528	1.2680	0.2610	0.783	0.1422	0.8939	0.8939
Heat shock	1.3089	0.2394	0.4788	0.1463	0.8889	0.8889	-1.2398	0.2619	0.7857

TABLE S4. Statistical analysis on vital activities between larvae that ingested cells containing *diap1-dsRNA and cells containing the vector only**

Treatment	Consumed leaf area				Body weight	
	0–24 hrs		24–48 hrs			
	t-value	*P*-value	*t*-value	*P*-value	*t*-value	*P*-value
EtOH	3.5773	0.01091	12.459	8.210E-06	16.175	9.433E-06
MeOH	4.3424	0.004016	16.689	0.0003703	11.94	4.205E-05
Heat shock	2.7042	0.03103	8.5303	8.937E-05	7.9661	0.0005294

5’- GGTAAATGTCATGGTTATGCAAGGAAGTAACCATCCCGTGTTTATGTTTATATTACTAGTTCGTTTCAATTACTCTAAAATTTGTA
GTGGATATAATCAAGATAATTGTTCTGGCTTTACGATTTCTGCAACTCATAAGTTTTAAACCTCGCATTCGAAAATGGTTCCACCA
GTAGAAGTATTGTCTTATCCAAGCTCGACTAGGAAGTTTGGTAACGGACTTGATTTGAAGAATATGCCCGCAGCAGTAAGTGTGAA
GAGAAATATAAATCATACCATAGGAAAAGACACTGGCGATAATGGTTGTTCCTTTTTGAATCTAACTCCACCCGCAAATTTATTGG
CTACGATCGAGGGACGCCTGAAGACGTACAAAAATTGGCCCAACAAGAATATAGATCCCCAGAAGTTAGCGGCCGCCGGCTTTTTC
TATTCTGGAAAAACTGACATCGTCGAGTGTTTCAAGTGCGGTATCAAGGGACACAACTGGTTGTTGAACGACGATCCAATGGAAGA
TCACAAAAAATGGAATAGGAATTGTTCTTTTGTAAGAGAAAACGCACCCGAAGAAAATAACGTCCCACAAACTGGCACCGGTAGTG
ATTATTGTGGCAATTTAGACGTCGTAACCTCACAATATACAGTCAGCGAAGAACCTGGAGATTATTATCGTAACTTGGGTGTGGAC
ATCTCTCCGTTCTTGCAAACTGGCGCTAAAACGAATCAGTCCGAGGGTCATAACCTGGAGGGGTTGTTACTAAGAACGAGGAAGGG
CCCAAGTCACCCAGATCAGATCATTTACGAGCGTAGAGTGGCGACATTCGCGAATTGGCCCAAGTCCTTGAAACAGAAACCCACGG
ACTTGGCGGCCGCAGGCTTTTACTACCTCGGAATCGGCGACCAGACGTTGTGCTTTTACTGCGGCGGCGGTCTGAAAGATTGGGTC
GAAGAAGACGATCCGTGGGAACAGCACGCGCTTTGGTTCCCCCAGTGTAATTATCTATTATTGAAGAAAACACCCGCTTTCGTCAA
AGACGTCCAAGAAAAACATAAAGGCGATTTGTCGTCATCCAAGCAAAACGAGACCGAAGTGGTAGCAAGTAGTAGCAGTAGTCACA
ACTCCAAAGAATCTCCAAGTGCGGTGGTAGAAGAGCGAGAAAGAAACAACGCAGAGGAAAGCTCGACATTATGCAAAATATGTTAT
AAAAATGAATTGGCTGTTGTATTTCTACCTTGCGGTCATATGGTAGCTTGTGTAGATTGTGCATCAGGATTAAAAGAATGTGCTAT
TTGCCGTAAAGAGATCCAAGCGAATGTTCGAGCCTTTTTGTCATAGTTCGCGAACAGTTAACAGTAACAGTTACTGCTTGACCACA
CTCATTTTCAAAGAAGTATGGTCGAATTATGTCTCCAGCTCAAAATCACCACCAAACAGTGACACGTTGTGGATGCATTTGTGTTT
CGACAATTACAGGTGGGTTCTCAGAACCCTAAAAGTGGCAGTTTTGGCGATTAACAAACCCATCTAGGTGAAAATGTGCTGCGTCG
CTCACGAAGATTTTGCTCGAAATACAGCATTCCCTTTTGATGCCGTATGTTGCAAACTTTCTTCACTGTGCATGGGCAAAAGGCTT
CAGTTGTTGTGGTTATTGAATTTGGCAAGCATGAGGACACAGATCTTTACTGATTATACGCTGTAGAGAGCTTCTTGAAATTTGAA
ATTCTTGTCCACGACGTCGAATTGAGATTCCTGGATTGTCACTGACACTTCGACGTACTACTGTGATTTTGACATTTGGACGACCA
TTGTATATGACGTATACAACGGATCCTGTCTCCCTGAATTCTTCATCTCTTCACAGTTGACGAAGTTAAAACACTATTCCGACAAC
ATTTCGAATGAAATTTTAGAACTGCTCTGCCAAGCTTTCATTATTTTTGAAATATTGTTCAATAATGAAGTAACGTTGTTCTATCG
TGTAATGTTCCATTTTTAATAACCTTAAACTGTC -3’

Fig. S1. DNA sequence of *diap1*-cDNA from *H. vigintioctopunctata*. The coding region of *diap1* is underlined. The DNA region used for production of *diap1*-dsRNA is indicated in red, and the target region amplified by qPCR is shaded in yellow. The regions corresponding to the sequences of the DNA primers used for qPCR are indicated by bases in cyan.

[Refs: Niimi T, Yoshioka H, Sato Y (2015) Insect pest control method. US. Patent US 9051569B2. (Date of patent: June 9, 2015); Y. Chikami et al. (2019) bioRxiv, doi: https://doi.org/10.1101/737643]

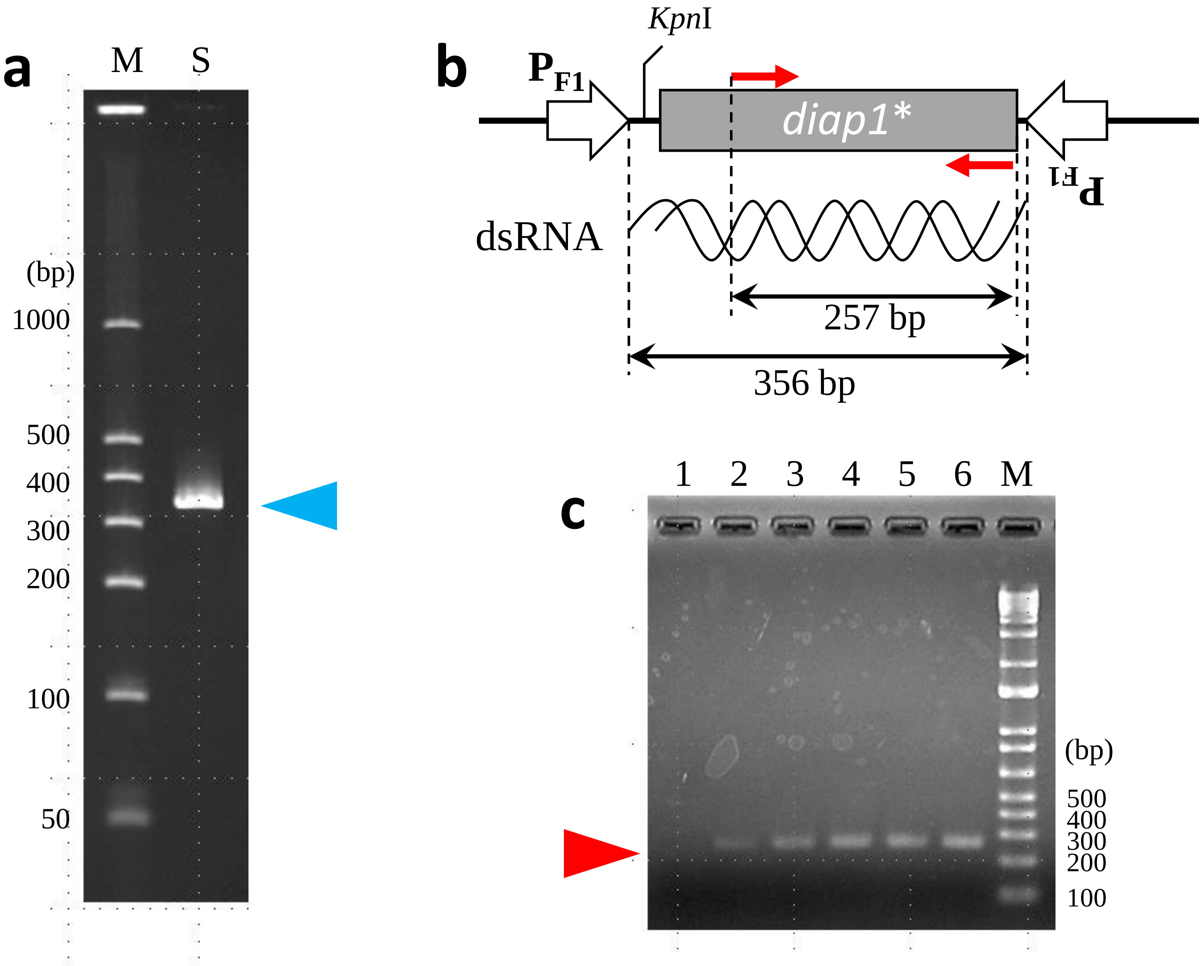

Fig. S2. Identification of *diap1**-dsRNA produced in *C. glutamicum* 2256Δ*rnc*/pVH2-HvIap-1 by RT-PCR. **a** Evaluation of the target RNA purified from total RNA of the producer cells. The purified RNA electrophoresed on 6% PAGE is indicated by a blue arrowhead. Lanes M, dsRNA marker; S, the target RNA sample. **b** Diagram showing the positions of primers (indicated by red arrows) used for RT-PCR. The length of the DNA fragment expected to be amplified by the RT-PCR is 257-bp. P_{F1}, F1 promoter. **c** DNA fragments amplified by RT-PCR. Red arrowhead indicates the expected DNA size. Amounts of template RNA for RT-PCR: 0 ng (lane 1), 0.001 ng (lane 2), 0.01 ng (lane 3), 0.1 ng (lane 4), 1 ng (lane 5), and 10 ng (lane 6). Lane M, DNA size marker. DNA was electrophoresed on a 2% agarose gel.

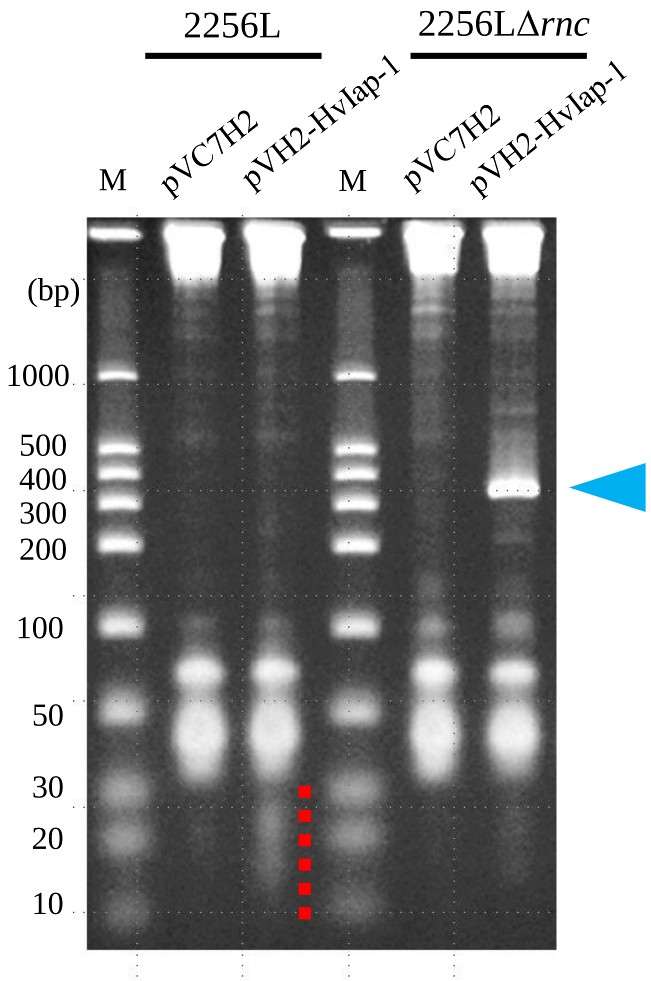

Fig. S3. Effect of *rnc* deficiency on *diap1**-dsRNA accumulation in *C. glutamicum* cells. The total RNA fraction from each strain indicated at the top of the gel was subjected to PAGE. Lane M indicates dsRNA size markers. A blue arrowhead indicates the position of *diap1**-dsRNA, and a red dotted line indicates some short nucleic acids which seem to be degradation products of *diap1**-dsRNA.

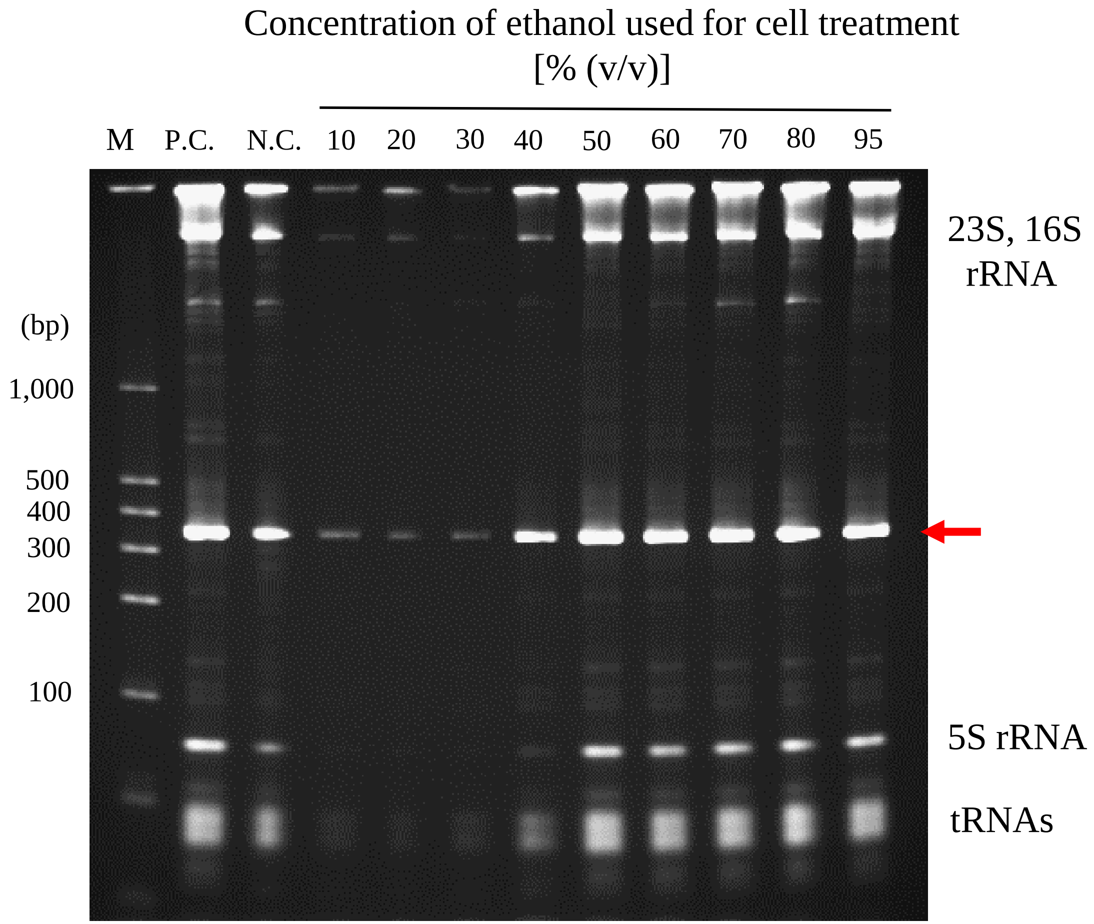

Fig. S4. Stability of *diap1**-dsRNA in *C. glutamicum* cells treated with various concentrations of ethanol. *C. glutamicum* cells accumulating *diap1**-dsRNA were treated with ethanol at the indicated concentrations. After treatment, the cells were collected as a cell pellet by centrifugation. The treated cells were allowed to stand at 20˚C for 24 h, then the total RNA was extracted from the bacterial cells and analyzed by 6% PAGE. The position of *diap1**-dsRNA is indicated with a red arrow. Lane P.C., without treatment with ethanol, RNA Protect Bacteria Reagent was immediately added to culture broth, and then total RNA fraction was extracted from the bacterial cells. Lane N.C. indicates total RNA from cells treated with phosphate buffer without ethanol. Lane M indicates dsRNA size markers.

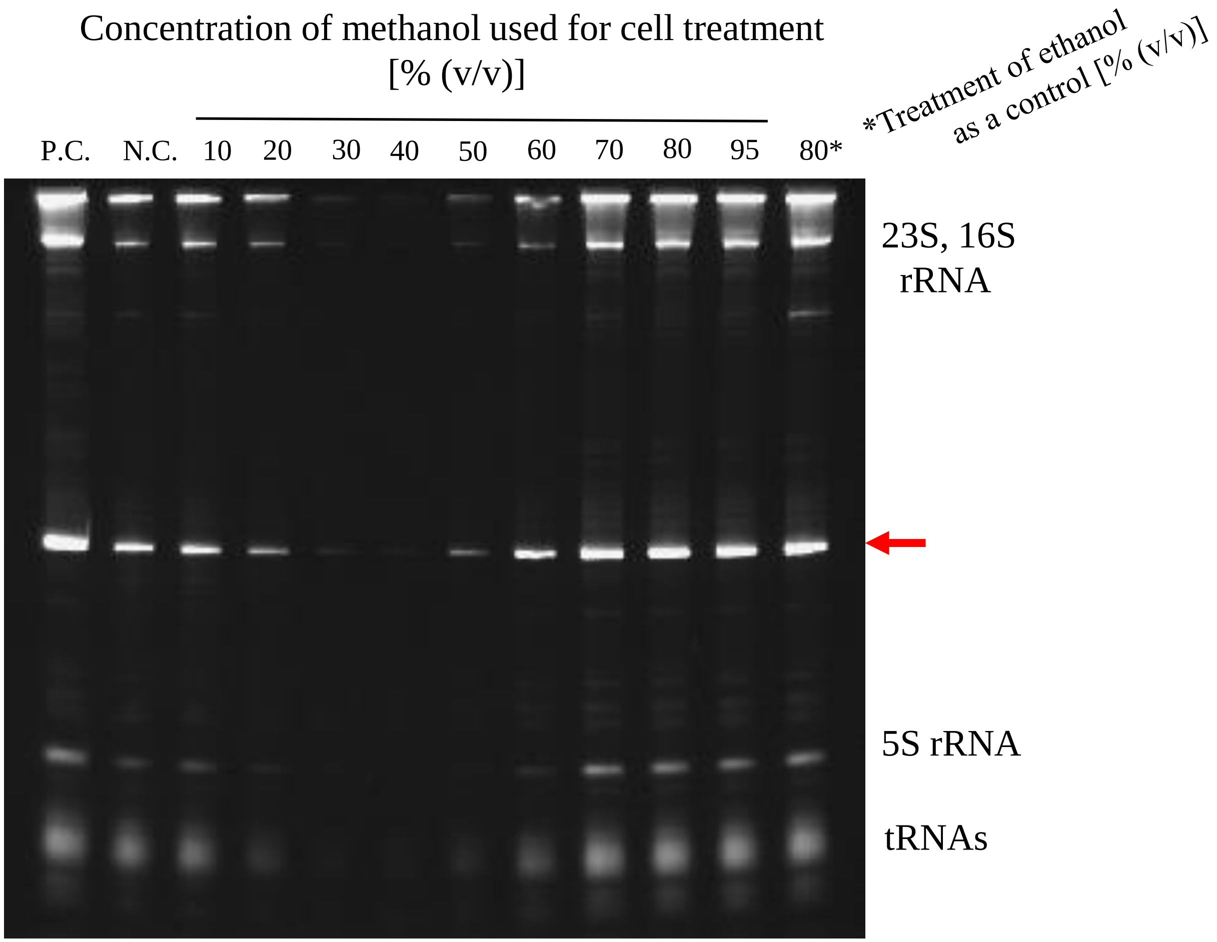

Fig. S5. Stability of *diap1**-dsRNA in *C. glutamicum* cells treated with various concentrations of methanol. *C. glutamicum* cells accumulating *diap1**-dsRNA were treated with methanol at the indicated concentrations. After the treatment, the cells were collected as a cell pellet by centrifugation. The treated cells were allowed to stand at 20˚C for 24 h, then the total RNA was extracted from the bacterial cells and analyzed by 6% PAGE. The position of *diap1**-dsRNA is indicated with a red arrow. Lane P.C., without treatment with methanol, RNA Protect Bacteria Reagent was immediately added to the culture broth, and then total RNA was extracted from the bacterial cells. Lane N.C. indicates total RNA from cells treated with phosphate buffer. Lane 80* is the total RNA fraction from cells treated with 80% (*v*/*v*) ethanol, as a reference. Lane M indicates dsRNA size markers.
